# Editorial: Modeling the immune system: from PK/PD to systems immunology

**DOI:** 10.3389/fimmu.2026.1800899

**Published:** 2026-02-17

**Authors:** Lucía Alfonso-González, Francisco J. Fernández, M. Cristina Vega

**Affiliations:** 1 Centro de Investigaciones Biológicas Margarita Salas (CIB-CSIC), Consejo Superior de Investigaciones Científicas, Madrid, Spain; 2 Abvance Biotech SL, Pharmacokinetics, Pharmacodynamics and Drug Metabolism (PPDM), Madrid, Spain

**Keywords:** biomarker, immune system, immune therapy, PK/PD, quantitative modeling, systems immunology

Understanding and ultimately controlling immune function takes much more than listing cell types, cytokines, and receptors. It requires quantitative ways of thinking and modeling that can capture the immune system as it really is: a highly complex, specialized, and dynamic network that operates across multiple scales – molecules, cells, tissues, and time. At the same time, these models need to remain understandable to immunologists so they can guide experiments, identify biomarkers, and support therapeutic decision-making.

This Research Topic was designed to help bring together two traditions that are increasingly overlapping: pharmacometrics and quantitative systems pharmacology (PK/PD, semi-mechanistic and mechanistic models), and systems immunology (data–driven and network-based approaches that integrate multi-omics and prior knowledge into coherent, testable frameworks).

The Research Topic brings together conceptual overviews, methodological advances, and applied case studies. It includes nine articles: eight original research papers and one review. Among the original research contributions, seven present computational models focused on specific aspects of the immune system, and one focuses on instrumentation and biomarker discovery. The review article examines how artificial intelligence and systems biology are reshaping modern immunology. Collectively, these contributions illustrate a central message: progress in immunology will accelerate when we treat immune responses as dynamic, multilevel systems that can be quantified with models, tested against data, iteratively refined, and ultimately used to predict how the system will respond to perturbations such as drugs, infections, or combination therapies.

Alfonso-González et al. offer a concise roadmap to the methodological “pillars” of systems immunology, highlighting how network pharmacology, artificial intelligence, and quantitative systems pharmacology can be combined to identify biomarkers, optimize therapies, and support drug discovery in inflammatory, autoimmune, and infectious diseases. The review does not ignore real-world constraints. It emphasizes challenges such as data quality, validation, interpretability, and regulatory requirements that must be addressed before these methods can have a genuine clinical impact.

Hong and Park propose “multi-physiology modeling” as a unifying paradigm for precision immunotherapy. They argue for integrating omics-driven systems immunology with dynamical modeling and pharmacometrics to represent cross-scale interactions and patient-to-patient heterogeneity. Their perspective underscores the growing need for modular, interoperable modeling frameworks that integrate multiple interacting agents and phenotypically diverse cell populations, linking single-cell and spatial data to mechanistic simulations to support individualized predictions of therapeutic outcomes.

Reneaux et al. broaden the scope of systems immunology by modeling immune-neurophysiological coupling in depression. In their framework, inflammation drives serotonergic dynamics and brain circuit activity. Their model suggests that treatment efficacy depends on baseline disease severity and that, in severe cases, a combination of anti-inflammatory and antidepressant strategies may be necessary to restore key variables to healthier ranges. This serves as a concrete example of how mechanistic models can generate clinically testable hypotheses about combination therapies.

Several original studies show how quantitative models can directly inform clinical questions. Huang et al. develop a QSP model to analyze combination therapy with camrelizumab and apatinib in advanced hepatocellular carcinoma. Using virtual patient simulations, they identify baseline immune predictors and explore dose adjustment strategies that preserve therapeutic benefit. Their work illustrates how mechanistic modeling can connect pharmacokinetics, immune state variables, and clinical trade-offs, enabling in silico exploration of treatment strategies.

Two contributions focus on measuring and interpreting humoral immunity. Kovács et al. introduce a dual-titration microspot immunoassay designed to yield truly quantitative serological readouts. By varying both antigen and serum antibody concentrations and fitting the resulting binding matrix, the method estimates affinity constants and antibody concentrations for different immunoglobulin classes. This yields parameters that can be directly incorporated into downstream systems analyses.

Kelbauskas et al. investigate the heterogeneity of antibody repertoires in Lyme disease using random peptide arrays and machine learning. They document substantial inter-individual variation and show how high-dimensional, unbiased antibody binding profiles can be used both to distinguish between groups and to trace signals back to biologically meaningful targets. This study serves as a compelling example of how modern machine learning workflows can be paired with experimental platforms to probe immune diversity.

Methodological advances in antibody modeling are addressed by Greenshields-Watson et al., who examine whether deep learning-based structure prediction can extrapolate beyond known canonical forms of antibody CDRs. Using large-scale predictions from ABodyBuilder2, the authors show that most predicted loops map to known structural space, with limited ability to generate out-of-domain conformations; importantly, they also demonstrate how even small amounts of relevant training data can restore predictive performance. The work provides a large, well-structured set of resources for the community.

Two network-oriented studies demonstrate how curated knowledge graphs and interaction maps can support mechanistic reasoning and translational discovery. Tang et al. present HUMPPI 2022, a human protein-protein interaction network used to examine the network positions of immune-related genes, patterns of virus-host targeting, and the influence of gene “age” on network topology. Their framework provides both concentrations of antigen-bound antibodies and estimated binding affinities, offering a valuable tool for immunological characterization and modeling. Their analyses also reinforce a recurring theme in systems biology: immune genes tend to be centrally located and pleiotropic, and viral targeting strategies can often be understood in terms of network architecture.

Finally, Niarakis et al. describe the COVID-19 Disease Map (C19DMap), a large-scale initiative to encode SARS-CoV-2-host mechanisms in a computable format and link these representations to bioinformatic pipelines for target identification and drug repurposing. This work shows how interoperable mechanistic content, combined with AI-assisted text mining and the integration of omics data, can accelerate hypothesis generation and help place candidate therapeutics within explicit pathways and cellular processes, with the ambition of ultimately tailoring these insights to individual patients.

Taken together, these contributions showcase the transformative potential of mathematical and computational modeling in immunology. By simulating and analyzing the complex interactions among biological components, researchers are advancing our understanding of immune mechanisms and host-pathogen interactions while opening new avenues for therapy. Deployed in this integrated way, these methods are already driving high-impact discoveries and equipping immunology with powerful tools to tackle some of its most urgent challenges.

